# Therapeutic implications of osteoprotegerin

**DOI:** 10.1186/1475-2867-9-26

**Published:** 2009-09-12

**Authors:** Sofia Fili, Maria Karalaki, Bernhard Schaller

**Affiliations:** 1 Medical School, National and Kapodistrian University of Athens, Athens, Greece; 2 Medical School, University of Oradea, Romania

## Abstract

Osteoprotegerin (OPG), a member of the tumor necrosis factor (TNF) receptor superfamily, contributes determinatively to the bone remodeling as well as to the pathogenetic mechanism of bone malignancies and disorders of mineral metabolism. There is additional evidence that OPG can promote cell survival by inhibiting TNF-related apoptosis-inducing ligand (TRAIL)-induced apoptosis. A number of recent *in vitro, in vivo* and clinical studies have defined the role of the RANK/RANKL/OPG pathway in skeletal and vascular diseases. These works were the milestone of the deep understanding of the mechanism of OPG. This review provides an overview of the potential innovative therapeutic strategies of OPG in metastatic breast and prostate carcinoma, multiple myeloma, postmenopausal osteoporosis, glucocorticoid-induced osteoporosis and rheumatoid arthritis. Special reference is given to the increasing evidence that RANKL and OPG may link the skeletal with the vascular system.

## Introduction

The key factor and dominant mediator of osteoclast differentiation, activation and survival is the Receptor Activator of NFkB Ligand (RANKL), which binds to its receptor (RANK) on osteoclast precursor cells and controls osteoclastogenesis and bone resorption. Osteoprotegerin (OPG), a secreted TNF receptor member that functions as a decoy of RANKL, regulates negatively this interaction [1-3]. Thereby inhibits osteoclast development and activation [1-4]. Understanding these molecular mechanisms of bone metabolism is crucial for developing novel drugs for treating such diseases [5]. Several studies in a variety of animal models have proved not only the dominance of RANKL blockade with OPG in inhibiting osteoclastogenesis and bone resorption, but also the role of deregulation of the RANK/RANKL/OPG system in the pathophysiology of multiple bone remodeling disorders such as osteoporosis, glucocorticoid-induced bone loss, erosive arthritis including rheumatoid arthritis, hypercalcemia of malignancy, Paget's disease of the bone and bone metastasis [6]. Moreover, clinical studies in post-menopausal women have shown that OPG can reduce the bone turnover [2,3]. These different experimental and clinical studies shed light on the fact that designing novel drugs that target RANKL-RANK and their signalling pathways in osteoclasts could potentially revolutionize the treatment of many diseases [6].

Except from its ability to inhibit osteoclastic activity, OPG seems also to play a key role on cell survival, via its interaction with TNF-related apoptosis-inducing ligand (TRAIL) [7,8]. TRAIL is a member of TNF superfamily. It is secreted by monocytes and seems to transduce apoptotic signals. More specifically, TRAIL binds to at least two membrane receptors (DR4 and DR5) and activates the death signaling pathway common to the TNF- family [7,8].

**Figure 1:**
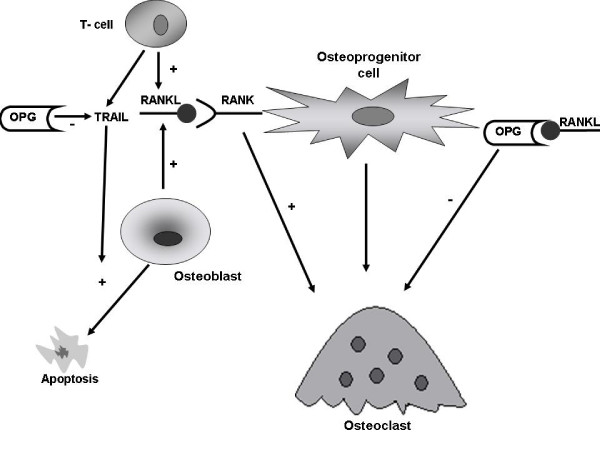
**The OPG/RANK/RANKL system and its interaction with TRAIL**. RANKL binds to RANK on osteoprogenitor cells and controls osteoclastogenesis and bone resorption. OPG regulates negatively this interaction and therefore inhibits osteoclast development and activation. TRAIL is being secreted by monocytes and is responsible for their ability to induce the apoptotic mechanisms of tumor cells. OPG facilitates the survival of prostate cancer cells due to its ability to bind to and inhibit the TRAIL death-activating receptors.

To date data show that OPG is a soluble receptor for TRAIL. Emery *et al *in their study have long demonstrated that OPG can bind to TRAIL *in vitro*[9]. Perhaps this interaction inhibits the binding of TRAIL to its death-activating receptors and therefore provides cells that produce OPG with a survival advantage [1,9]. More recent studies have also shown that *in vitro *OPG (through the inhibition of TRAIL- induced apoptosis) could be a survival factor for prostate cancer cells [10], breast cancer cells [11] and multiple myeloma cells [12] (figure 1).

Despite the fact that till now there are not many data about OPG-TRAIL interaction *in vivo*, the exact regulating of the concentration, timing and location of expression of OPG and TRAIL in tissue microenvironment might determine the effects of their interactions *in vivo *[8]. OPG represents therefore a novel therapeutic option based on the remarkable advances occurring in the fields of cellular and molecular biology as applied to research in molecular medicine [13]. In the present review we want therefore to present the therapeutic potential of OPG for different diseases based on its molecular mode of action.

## Bone malignancies

In patients with cancer, metastasis represents a major cause of morbidity and mortality. Bone is nowadays among the most frequent sites of hematogenous tumor metastasis in human body, representing around 20-40% of all sites of metastasis. Clinically, bone metastases cause severe complications (such as bone pain and fractures) and usually indicate a worse prognosis for the patient. Histologically, bone metastases are traditionally divided in osteolytic (where bone resorption is stimulated) and osteoblastic (where new bone formation is enhanced). However, today it is well known that in most osseous metastasis it's rather the balance between bone lysis and formation that is deregulated, leading to an unbalanced bone remodeling process and therefore to mixed lesions [1].

Bone pain, which represents one of the most severe complications of bone metastases, can be minimized by OPG. Honore et al. have proved that OPG blocks behaviors indicative of pain in mice with bone cancer [14]. Excessive tumor-induced bone destruction is involved in the generation of bone cancer pain and OPG by reducing bone resorption may provide an effective treatment for this common human condition [14].

In treating of metastatic bone disease, the primary goals are the preventing a bone lesion from developing and limiting the progression of an established bone metastasis. However, the currently available therapies for bone metastasis such as bisphosphonates, radiotherapy, radiopharmaceuticals and surgery focus only on symptomatic management [15,16]. However, biophosphates - for example - block not only bone resorption but also tumor-cell mitosis and stimulate tumor-cell apoptosis. Bisphosphonates also alleviate the above mentioned bone pain [17,18]. Additionally, OPG- like peptidomimetics are also effective in suppressing bone resorption in patients with tumor-induced bone disease [19,20]. Anti-RANKL antibodies, such as denosumad, represent a new class of RANKL inhibitors that are characterized by high specificity for RANKL and so they prevent RANKL from binding to its receptor and stimulating osteoclasts resulting in the reduction in bone resorption [21,22].

### Prostate cancer

Prostate cancer is among the malignancies that have great avidity to bone [1,5]. OPG has been shown to be secreted by prostate cancer cells [10]. It can bind to RANKL and prevent osteoclastogenesis [23,24]. It is also estimated that *in vitro *OPG can protect the tumor cells from apoptosis, via its ability to inhibit TRAIL (TNF- related Apoptosis Inducing Ligand) and the apoptotic mechanisms it activates [11,10,25]. On the contrary *in vivo *OPG was shown to inhibit the survival of prostate cancer cells in bone [23,26]. Recent *in vivo *studies also show that OPG can inhibit RANKL-induced cancer cell migration to the bone. A possible mechanism is that RANKL can induce prostate cancer cell migration by binding to RANK which is expressed on the tumor cells [27,28]. OPG binds to RANKL and blocks its interaction with RANK inhibiting RANKL-mediated cell migration to the bone [28]. This effect is not observed in other tissues, except from the bone. This tissue-specific inhibition of prostate cancer cell migration might be additionally partly due to other factors related to the local bone microenvironment [27,28]. However, it is not yet clear whether this ability of OPG to inhibit the development of osseous lesions could have any clinical implications in the treatment of patients with tumor-related bone disease [26].

In clinical level, several studies have shown that serum OPG concentrations are significantly higher in patients with prostate cancer [29], and even higher in those with advanced disease [30], suggesting that prostate cancer cells over-secrete OPG. Such data indicate a potential role of serum OPG as a surrogate marker of the disease progression or early relapse in patients with prostate cancer.

### Breast cancer

Concerning the role of OPG in breast cancer metastases to bone, it has been shown that breast cancer cells can produce OPG *in vitro *[11]. This seems to be very important, as OPG can protect breast cancer cells from undergoing TRAIL-induced apoptosis [11,21,31]. On the other hand, OPG treatment in animal models of breast cancer inhibited the development of osseous lesions [32,33] and the growth of cancer cells in the bone [32]. This was a result of the treatment with high dose of OPG (up to 25 mg/kg) as controlled after 4 weeks with a Faxitron X-ray system and histological analysis [32]. Clinical studies do not yet exist. But these experimental data indicate that OPG could perhaps be a potential therapeutic option for the treatment of bone lysis observed in patients with metastatic breast cancer, but further studies are essential to establish this role.

### Multiple myeloma

Multiple myeloma is a malignant clonal proliferation of B-lymphocyte- derived plasma cells, characterized by its ability to develop osteolytic bone lesions, via the induction of osteoclastic bone resorption [34].

Recent studies have revealed that myeloma cells do not express OPG, but via intracellular interactions with osteoblasts and Bone marrow stem cells (BMSCs) can down- regulate the OPG released from these cells [35]. Moreover, they can bind, internalize and degrade OPG *in vitro *[34]. This process leads to decreased OPG concentrations in bone marrow micro-environment and therefore to increased osteoclast activation via the RANKL pathway [34]. In the 5TMM murine model of multiple myeloma, treatment with OPG protected the mice from myeloma- induced bone disease as measured after 4 weeks by the radiographic Faxitron x-ray system [35,36]. Recent experimental data also suggest that multiple myeloma cells enhance the expression of RANKL and reduce that of OPG in their microenvironment [37]. This seems to be a result of interaction between multiple myeloma cells and BMSCs indicating that multiple myeloma cells can cause an imbalance of OPG/RANKL axis and that this dysregylation is involved in the pathogenesis of multiple myeloma disease [37]. On the other hand, OPG is shown to prevent TRAIL-induced apoptosis in human myeloma cell lines [9]. It is therefore intriguing to investigate why the down-regulation of an anti-apoptotic factor may take part in the development of myeloma bone disease.

Clinical studies have shown that the serum concentrations of OPG are lower in patients with multiple myeloma, and even lower in those with evidence of bone lesions suggesting that OPG plasma concentrations may be related with the degree of osteolysis [38]. In a phase I clinical trial, the treatment of patients with a single-dose of OPG was shown to inhibit significantly bone lysis, as 56 days after the treatment the levels of bone resorption markers (and especially of urinary N- telopeptide/creatine) were decreased [10,39].

These clinical and also experimental data strongly suggest that deregulation of the OPG expression is involved in multiple myeloma. OPG could be not only a possible surrogate marker for multiple myeloma in the management of patients with, but also a potential therapeutic target for the treatment of the multiple myeloma [34,40].

It should also be mentioned that during the last years there has also been increasing interest in the role of Dickkopf 1 (DKK-1) in multiple myeloma. DKK-1 is a soluble inhibitor of Wnt signaling pathway, which prevents osteoblast differentiation. DKK-1 can also inhibit the expression of OPG in osteoblasts [41]. Therefore, DKK-1 both directly and indirectly enhances the osteoclastic activity in bone microenvironment. DKK-1 is secreted by myeloma cells and is over-expressed in patients with advanced myeloma bone disease, as shown by measurement of DKK-1 levels in both bone microenvironment and serum [42]. Overall, these data suggest that DKK-1 might partly be responsible for bone lysis observed in multiple myeloma disease [41,42].

### Hypercalcemia of malignancy

By binding and neutralizing RANKL, OPG diminishes osteoclast formation and activity resulting in blocking bone resorption in animal models of hypercalcemia of malignancy [43-45]. Studies have indicated that OPG, given either at the onset of hypercalcemia or after it had occurred (hypercalcemia over 3-5 days), blocks tumor-induced bone resorption and hypercalcemia and therefore leads to normalization of blood calcium within 48 hours [43-45] suggesting an effective method for curing this paraneoplasmatic syndrome. OPG can effectively reverse hypercalcemia in less than 3-5 days in most patients [44].

## Disorders of mineral metabolism

As it has been already mentioned, OPG regulates negatively the interaction between RANKL and its receptor RANK and therefore inhibits osteoclast development and activation. This is the main pathway that explains that the RANK/RANKL/OPG system contributes significantly to the pathophysiological mechanism of several bone disorders by regulating osteoclastogenesis [2] and may represent the key factor for the discovery of the therapeutic properties of OPG for these diseases.

### Postmenopausal osteoporosis

The discovery of the determinative contribution of the RANK/RANKL/OPG system in the osteoclastogenesis indicates its role in the pathogenesis of postmenopausal osteoporosis [2,46]. Recent studies have shown that the up-regulation of RANKL on bone marrow cells induced by estrogen deficiency in postmenopausal women is responsible for the bone resorption [47]. By blocking the interaction of RANKL with RANK in vivo, OPG reduces the osteoresorptive results of this signaling pathway [48]. Furthermore, several investigations using a mouse ovariectomy model of estrogen deficiency-induced osteoporosis concluded to the fact that OPG not only prevents bone loss but also increases bone mineral density leading to osteopetrosis when over-expressed [48-50]. It has been also shown that a single injected dose of OPG in post-menopausal women causes rapidly and profoundly reducing bone turnover after 12 hours as measured by the decreasement of the biochemical markers, urinary N-telopeptide (NTX) and deoxypyridinoline (DPD), which are stable collagen degradation products [51].

Low-dosage intermittent parathyroid hormone (PTH) treatment has an anabolic effect on the skeleton and this ability makes PTH a promising therapeutic treatment for osteoporosis. However, intermittent administered PTH also stimulates bone resorption and therefore can cause hypercalcemia, which can be inhibited by co-administration of PTH and antiresorptives [48,52]. A significantly greater and more rapid increase in bone mineral density is proved by OPG-PTH co-treatment rather than administering the agents separately [49]. Therefore the combination of OPG and PTH represents an effective therapeutic approach to treating or reversing severe osteoporosis in humans. A further advantage of PTH combined with OPG is the inhibition of the hypercalcemic effects of PTH treatment [48,52].

### Glucocorticoid-induced bone loss

Osteoporosis is a well known side-effect of long-term glucocorticoid treatment, but the precise underlying mechanism remains unclear. Recently, it has been proved that glucocorticoids affect the RANK/RANKL/OPG pathway by stimulating RANKL expression by osteoblasts and inhibiting OPG synthesis and thereby resulting in osteoclast differentiation, activation and survival [53-55]. These findings explain that glucocorticoid-induced bone loss develops rapidly because of the combination of increscent of bone resorption and suppression of bone formation [2,53-55], suggesting that the reversal of this bone loss is possible by treating with OPG. These findings may open new therapeutic options to effectively treat the glycocorticoid-induced osteoporosis.

### Rheumatoid arthritis

Rheumatoid arthritis is a chronic disease which is characterized by progressive synovial inflammation and joint destruction. Recently, it has been demonstrated that the RANK/RANKL/OPG system participates in the pathophysiological mechanism of focal and generalized bone loss in rheumatoid arthritis as it is described below [2,4]. Osteoblastic-stromal cells, synovial fibroblasts and activated T-cells are important inducers of RANKL expression leading to loss of the RANKL/OPG-balance, resulting in the osteoclastogenic proceeds in erosive arthritis [4,56]. Interestingly, studies have shown that higher serum levels of OPG and RANKL are present in patients with rheumatoid arthritis than in healthy people, but the ratio of OPG/RANKL is similar [40, 41, 42]. These data conclude to the fact that anti-TNF therapy normalizes the OPG/RANKL balance stimulating the bone erosion in arthritis [56-58].

In a T-cell-dependent model of rat adjuvant arthritis characterized by severe joint inflammation, bone and cartilage destruction and crippling, blocking of RANKL through treatment with OPG at the onset of disease prevents bone and cartilage destruction but not inflammation [59]. These results show that both systemic and local T-cell activation leads to RANKL production and therefore bone loss. This study of Kong et al. demonstrates nicely that bone remodelling and bone loss are controlled by a balance between RANKL and OPG [59].

### Fractures

Although the effects of OPG on fracture healing are not so clear, it has been proved that expression of OPG and RANKL in callus tissue is involved in the mechanism of endochondral resorption and remodeling [4]. The clinical study of Vinther et al. including 80 patients has demonstrated that although OPG treatment impairs remodeling of healing fractures and decreases the mechanical quality of the callus tissue, OPG does not influence the strength of the fractures in total [4].

### Renal osteodystrophy

In patients with uremia either increased or suppressed bone turnover can be observed [2]. However, secondary hyperparathyroidism is the most common type of renal osteodystrophy and is characterized by PTH-related bone loss. OPG serum levels are increased in patients with chronic kidney disease [2] and it might inhibit osteoclastogenesis and bone resorption induced by PTH in patients with uremia [60]. Therefore, OPG serum concentrations could possibly be used as surrogate markers of the progression of bone disease in patients with uremia, but further studies are needed to establish this role [2,61].

## Vascular Diseases

Several epidemiological studies have shown that osteoporosis and vascular calcification could be associated [2,62,63] and share many common risk factors [62]. Although the mechanisms responsible for this phenomenon are not clear yet [64], OPG is today believed to be an important mediator of it. More specifically, it is estimated that a deregulation of calcium allocation results in its movement from the bone to the endothelium of the vessels through mechanisms that involve OPG [62].

It is also interesting that endothelial cells (ECs) and vascular smooth muscle cells (VSMCs) produce and express all members of the OPG/RANK/RANKL-axis [62]. RANKL produced by ECs and VSMCs interacts with RANK- receptors on the surface of ECs and might participate in the process of atherosclerosis via mechanisms that are today under investigation. In a recent *in vitro *study, RANKL caused a dose-dependent increase of calcification in VSMCs, which was inhibited by OPG. The researchers hypothesize that RANKL binds to RANK and through the activation of an intracellular pathway leads to an increased production of bone morphogenetic protein 4 (BMP4) in VSMCs, which induces vascular calcification [65].

OPG is believed to antagonize RANKL actions in vessels as well [62]. However its role on vascular tissue is much more complicated, as its production can either be induced or down-regulated in response to various inflammatory mediators, and can therefore affect the process of vascular calcification in multiple ways [62].

### Vascular calcification

The actions of OPG have been studied in a number of animal models [62,66-68]. *In vivo *studies in OPG knockout mice indicate calcification of the aorta and renal arteries [62,66]. These are both sites where OPG is normally expressed [67]. These findings suggest that OPG produced in these arteries might protect them from early calcification and that its lack or deficiency might take part in pathological artery calcification [67].

Such a vascular protective role is also suggested by a study in which treatment by OPG could inhibit the vascular calcification induced by warfarin and vitamin D [68]. Warfarin causes calcification by inhibiting the vitamin K- dependent carboxilation of matrix Cla protein (MGP). Injections of OPG in rats treated previously with warfarin inhibited arterial calcification [68].

On the second animal model of this study, treatment with high doses of vitamin D caused extended calcification of the arteries in rats, which was also inhibited by injections of OPG [68]. The researchers suppose that the vascular-protective role of OPG in their animal models is a result of two possible mechanisms; on the one hand limited bone resorption and on the other hand direct promotion of the expression of calcification inhibitors (e.g. MGP) in vascular cells [68].

On the contrary, clinical studies have revealed that serum OPG concentrations are increased in patients with cardiovascular disease and may serve as an independent risk factor of cardiovascular events [63,69,70]. Moreover, its key- role in the regulation of the inflammation, proliferation and apoptosis of various cell types in tissues where it is expressed (e.g. the bone) indicate that OPG may also participate in such procedures in the vascular tissue as well [63]. Therefore, further researches and essential in order to establish a possible role of OPG not only as a marker, but also as a therapeutic target of cardiovascular disease.

## Conclusion

The RANK/RANKL/OPG system plays a determinative role in the pathophysiology of different bone and vascular diseases and increases our understanding of the contribution of OPG to the treatment of those disorders. However, greater insights into the healing mechanism of OPG concerning bone diseases would lead to new therapeutic approaches that limit or prevent such disorders. Therefore, further researches are indispensable, since questions related to the therapeutic role of OPG have to be answered, but OPG remains a breakthrough in the treatment of bone disorders.

## Competing interests

The authors declare that they have no competing interests.

## Authors' contributions

FS and KM have equally contributed to the elaboration of the review. SB was the senior author. All authors read and approved the final manuscript.
